# Comparing health system performance assessment and management approaches in the Netherlands and Ontario, Canada

**DOI:** 10.1186/1472-6963-7-25

**Published:** 2007-02-23

**Authors:** Ali R Tawfik-Shukor, Niek S Klazinga, Onyebuchi A Arah

**Affiliations:** 1 Department of Social Medicine, Academic Medical Center, University of Amsterdam, PO Box 22700, 1100 DE Amsterdam, The Netherlands; 2 Netherlands Institute for Health Sciences, Erasmus MC, PO Box 2040, 3000 CA Rotterdam, The Netherlands; 3 Center for Prevention and Health Services Research, National Institute of Public Health and the Environment, PO Box 1, 3720 BA Bilthoven, The Netherlands

## Abstract

**Background:**

Given the proliferation and the growing complexity of performance measurement initiatives in many health systems, the Netherlands and Ontario, Canada expressed interests in cross-national comparisons in an effort to promote knowledge transfer and best practise. To support this cross-national learning, a study was undertaken to compare health system performance approaches in The Netherlands with Ontario, Canada.

**Methods:**

We explored the performance assessment framework and system of each constituency, the embeddedness of performance data in management and policy processes, and the interrelationships between the frameworks. Methods used included analysing governmental strategic planning and policy documents, literature and internet searches, comparative descriptive tables, and schematics. Data collection and analysis took place in Ontario and The Netherlands. A workshop to validate and discuss the findings was conducted in Toronto, adding important insights to the study.

**Results:**

Both Ontario and The Netherlands conceive health system performance within supportive frameworks. However they differ in their assessment approaches. Ontario's Scorecard links performance measurement with strategy, aimed at health system integration. The Dutch Health Care Performance Report (Zorgbalans) does not explicitly link performance with strategy, and focuses on the technical quality of healthcare by measuring dimensions of quality, access, and cost against healthcare needs. A backbone 'five diamond' framework maps both frameworks and articulates the interrelations and overlap between their goals, themes, dimensions and indicators. The workshop yielded more contextual insights and further validated the comparative values of each constituency's performance assessment system.

**Conclusion:**

To compare the health system performance approaches between The Netherlands and Ontario, Canada, several important conceptual and contextual issues must be addressed, before even attempting any future content comparisons and benchmarking. Such issues would lend relevant interpretational credibility to international comparative assessments of the two health systems.

## Background

Both Ontario and The Netherlands have shown interest in health systems performance assessment and management through the development of performance indicators within supportive conceptual frameworks [1-7]. The two healthcare systems underwent significant reforms in 2006 that promise to produce, at lower cost, greater access to and better outcomes from healthcare than their previous policies do. Both systems aim to create new efficient healthcare systems that are equitable, patient-focused, results-driven, accessible and sustainable [8-10]. The respective Ministries of Health have created conceptually-sound performance indicator frameworks to actively measure, manage and operationalize the performance of their health systems, thereby linking performance measurement to ongoing policy and accountability processes. In an effort to promote common learning and best practise, policymakers from both constituencies expressed interest in learning from each other's performance

Both Ontario and The Netherlands have gone through great lengths to develop comprehensive health system performance assessment (HSPA) frameworks that avoid the theoretical, methodological and operational pitfalls of previous HSPA studies. We will illustrate how these national and provincial conceptual frameworks can be used to give a relatively objective picture of performance over time and between healthcare contexts. This comparative project evaluates how performance is assessed in two constituencies using differing regulatory regimes (Ontario's Beveridge and the Dutch Bismarckian systems). Such a comparative performance assessment study could provide valuable guidance for future attempts towards benchmarking.

The Canadians were among the first to realize the potential value of benchmarking efforts, spurred by the September 2000 First Ministers' Communiqué on Health that has resulted in the development of the Canadian Health Indicator Framework (CHIF) [11]. The CHIF has served as the pioneering comprehensive theoretical base for many modern national and international health system performance assessment frameworks, including that of The Netherlands and the OECD Health Care Quality Indicator (HCQI) project [5,6]. The province of Ontario has recently published its personalized Health System Scorecard (OHSS), an innovative and functional framework composed of nine strategic health system performance themes (dimensions), populated by a balanced set of 27 indicators. The themes are portrayed using a series of cause-and-effect linkages showing how the system ultimately "creates value" for the population [12,13].

The Dutch have also moved forward with the critical assessment of performance initiatives, and have focused on measuring the performance of their national health system. The Dutch Ministry of Health, Welfare and Sports (Ministerie van Volksgezondheid, Welzijn en Sport, or VWS) commissions the National Institute of Public Health and Environment (RIVM) to analyze such reports in an effort to translate the results of benchmarking analyses into effective policies [14]. In Dutch health policy a distinction is made between health and healthcare performance by the release of two separate 2006 national reports: the Dutch Health Care Performance Report (Zorgbalans) and the Public Health Status and Forecasts Report (PHSF, or Volksgezondheid Toekomst Verkenning). The Zorgbalans deals with management and performance information specific to health care (quality, access and cost of health care), whereas the PHSF report gives an overview of the public health perspective (health of the population). The former focuses on the production of effective and sustainable health care; the latter on a health system's ultimate goal: health [15]. The Dutch national health system performance conceptual framework, heavily based on the CHIF and US National Healthcare Quality Report, has been adopted as the theoretical framework of the OECD's HCQI project [16,5,6].

We compared health system performance methodologies in The Netherlands with Ontario, highlighting what conceptual, operational, and contextual policy factors must be taken into account when attempting future benchmark initiatives, and clearly illustrating the extent of the interrelations between the performance frameworks.

## Methods

Health system performance assessment in The Netherlands and Ontario was assessed in both locations during the period January to July 2006. We examined their conceptual frameworks, performance dimensions, indicator sets and embedded strategy bases using planning, management and policy documents published by the Dutch [8] and Ontario [9] Ministries of Health (OMHLTC), RIVM [17], the Canadian Institute for Health Information (CIHI) [18], and OECD [19]. Additional literature and data was retrieved using PubMed and the generic Internet search engine Google [20]. Our analysis of Dutch and Ontario HSPA was validated via emails and interviews with stakeholders representing OMHLTC's Health Results Team (HRT), the RIVM, and the University of Amsterdam Medical Center's (AMC) HSPA team.

Information detailing the key dimensions, indicators and strategy bases of health system performance was abstracted using a pro-forma, highlighting how and why they were selected, their robustness and validity, as well as any contrasts and commonalities between the two sets. Comparative descriptive tables and schematics were assembled to examine the interrelations between the performance assessment frameworks. A backbone 'five diamond' framework was developed to link the Dutch Zorgbalans healthcare performance matrix and Ontario Health System Scorecard.

Twelve participants representing policymakers and researchers from The Netherlands (3 particpants), Ontario (8 participants) and the United States (1 participant) attended a two-day workshop in Toronto (July 17–18, 2006) to discuss the findings, validate the comparative framework, and to extend relevant contextual policy factors to the study. The workshop was commissioned by the Canadian Institutes of Health Research (CIHR) and the Institute of Health Services and Policy Research (IHSPR) Community Development Funding Program.

## Results

### The Conceptual Frameworks

#### The Netherlands

**Figure 1:**
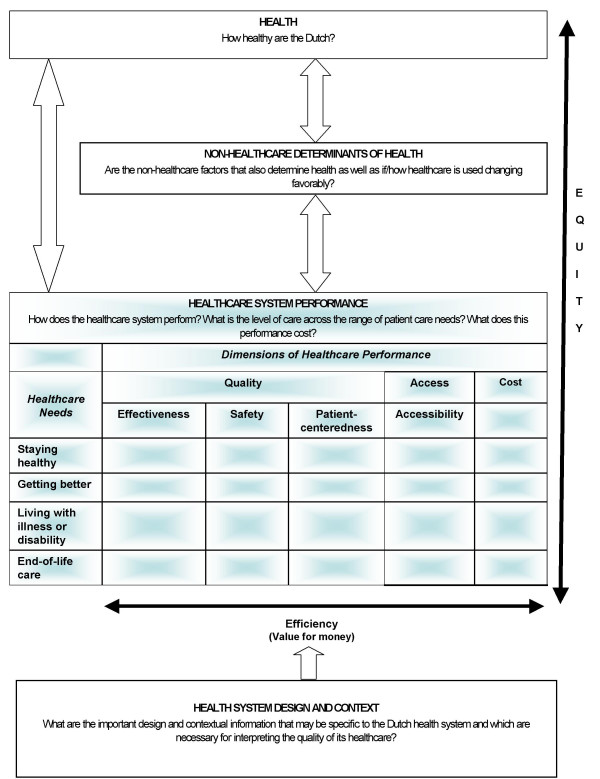
Conceptual Framework for Dutch National Health System Performance.

In January 2002, the Dutch Ministry of Health, Welfare and Sport (VWS) commissioned the RIVM with the development of a national performance indicator framework for the Dutch health system (Figure 1), and stressed the need to focus future efforts towards the participation in international benchmarking projects [3,10]. The conceptual framework governing the 2006 Zorgbalans and Dutch PHSF report focuses on the technical quality of healthcare, while keeping a broader perspective on health and its other determinants. The third tier of the Dutch framework (healthcare performance) is the basis of the 2006 Zorgbalans, OECD HCQI project, and is the focus of this paper. This tier differs from the CHIF in that it is composed of a matrix of healthcare performance dimensions (columns) by healthcare needs (rows). Dimensions of quality (effectiveness, safety and responsiveness/patient centeredness), access, and cost/expenditure are measured against healthcare needs (prevention, cure, chronic care, and palliative care).

The quality, access and cost dimensions are proposed to map into outcome, process and structure indicators, respectively [5,6]. However, the current access dimension is heavily outcome based.. The 2006 Zorgbalans is comprehensive, robust, and multidimensional, resulting in a set of 125 indicators that are recognizable, relevant and appropriate for their policymakers [15,21,22].

#### Ontario

The Canadian province of Ontario has developed a framework that is sensitive to its own key health performance and management issues. The Health Results Team (HRT) was created in September 2004 to implement several major innovative system-wide transformation initiatives, with information management at its core [12].

**Figure 2:**
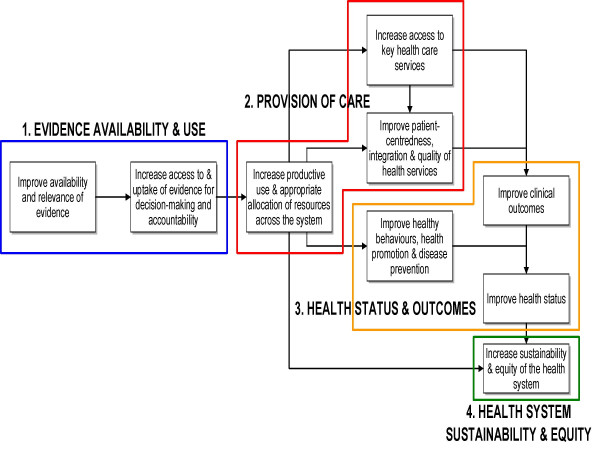
Ontario Health System Strategy Map.

To streamline information and improve data quality, the HRT have developed a provincial Health System Scorecard (OHSS) based on health system strategies, drawing on a few carefully-selected measures that convey the performance of the overall health system [12,13]. Through an iterative issue abstraction and strategy mapping exercise, the HRT reported a set of nine strategic goals (themes/dimensions) that best reflect the full extent of the health system's ongoing performance improvement initiatives, and are populated by a balanced set of 27 indicators relevant to health system renewal (Figure 2) [23,24].

The nine dimensions reflect both overall health system goals as well as current government priorities, are strategically linked to performance management, and fall within four key quadrants of performance: 1) Evidence availability and use, 2) Provision of care, 3) Health status and outcomes, and 4) Health system sustainability and equity. These quadrants form the core chapters of the 2006 Scorecard, providing an overall picture of performance in Ontario.

### Operationalizing Performance

#### The Netherlands

Strategic objectives, aims, and goals of the Dutch health system have been integrated as the sub-questions within the chapters of the Zorgbalans, all of which have been formulated to fit within the framework's matrix comparing healthcare performance with healthcare needs. The Zorgbalans' conceptual framework is composed of 15 topics (paragraphs) that coincide with many "system targets" of the Dutch Ministry of Health, Welfare and Sports [21,23]. Ultimately, such a structure is intended to enable researchers to provide policymakers the evidence-base they need to make appropriate policy actions; however, the framework was not designed to explicitly link performance information with health system management and strategy:

#### Ontario

The nine themes and four performance quadrants of the Ontario Scorecard mutually reflect overall health system goals as well as current government priorities. Ontario's Health System Strategy Map (OHSS) (Figure 2) articulates strategies for performance improvement through a series of hypothesized cause-and-effect linkages between the nine strategic themes, in order to demonstrate how the health system creates value for the population. [4,12,13,7]. Using the 9-themed Strategy Map, the framework can be cascaded down to the Local Health Integrated Network (LHIN) level, effectively linking performance measurement to accountability on various functional levels [4,7,23,24].

### Policy context

A CIHR-commissioned workshop was held in Toronto to better understand the higher-level contextual meaning behind the performance assessment frameworks. Stakeholders expressed interest in understanding how several independent contextual variables (for example,. regulatory regimes, state structures, funding systems, health system governance, performance reporting, quality incentives, budgetary cycle policies, funding formulas, decentralization and local health system autonomy, performance contracting, strategic purchasing) cause differences in health system performance in The Netherlands and Ontario. The roundtable discussion extended important contextual policy information into the study, further validating the results of the initial information collected. Findings from the workshop are summarized below in Table 1.

**Table 2:** Summary of policy and contextual factors

**Ontario**	**The Netherlands**
**Governance**	

• Defined as "stewardship" • Integration and decentralization management processes • Supply-driven management • System level accountability and multi-level budget allocation (federal/provincial/LHIN/project) through performance measurement • Public system to be sustained	• Defined as "system responsibility" • Regulated-market steering mechanism • Demand-driven management • System level accountability and transparency through performance measurement • Focus on suppliers and insurers • Private sector, public finance

**Operationalization/health system strategy**	

• OHSS strategy map conceptualizes strategy • Top-down steering mechanism → Government directs various health system actors (central role in system management, despite devolution of power) • Stewardship, regulation, goal setting, performance expectations	• Zorgbalans has 15 performance dimensions within 3 domains (quality, access and cost) • No harmonized mapping of strategy → 15 dimensions are categories of information, not strategy-based • No target setting • Steering mechanism → Government provides guidelines, but actors set the strategies ("system responsibility")

**Health system structure**	

• Currently undergoing decentralization/regionalization reforms • Local Health Integrated Networks (LHINs) • 14 geographical entities, ranging in size • Concentrated in southwestern Ontario • Roughly 500,000 inhabitants/LHIN	• Insurers have consolidated (from 100 to 5 currently operating, forming an oligopoly, working on economies of scale) • Obligatory basic insurance package within a competitive regulated market • Insurers contract providers (performance measures embedded in this process) • Equalization fund for the elderly and people with chronic disease is a driver of the strategic behaviour of the insurers. In addition, the Health Insurance Income Support Law (Wet op de Zorgtoeslag) compensates lower-income groups against increases in premiums • Hospital holdings created to increase market power • Providers regulated by an Inspectorate for Health Care (Inspectie voor de Gezondheidszorg (IGZ)) through layered inspection, using information management to target site visits

**Reporting structure**	

• Ontario Quality Council, Hospital Report (being discontinued and taken up by CIHI), development of electronic performance indicators, ICES Atlases (science-driven themes, not sector-based), Cancer Care Ontario, Frasier Institute, Conference Board of Canada reports, biannual Health Ministers Report, CIHI, Statistics Canada, Canada Quality Council and Ministry of Health Promotion	• RIVM (thematic reports, similar to ICES Atlases), National Cancer Institute (NKI), sector-specific reports, RIVM Public Health Status and Forecasts Report, Cost of Illness (2003) by disease category, Cost of prevention (2003), Health Report from Office of Statistics, Social and Cultural Planning Bureau, and Netherlands Institute for HSR reports (Nivel)

**Quality incentives**	

• Ontario Best Practise Registry (IHI model-based listing sector-specific best practices) • Provincial Performance Fund ($5 million CAD) for providers developing CQI projects with good return on investment	• National overseeing of quality control initiatives carried out by insurers. • – Initiative of the Dutch MoH comparing information on care, insurers, hospitals and medicine costs • College Toezicbt Zorgverzereringen (CTZ) was the specialized body that supervised social health insurance

**Budget cycle and funding formulas**	

• No direct link of funding formula with OHSS • Looking at pay-for-performance and other innovations • Public Sector Value (PSV) model linking performance framework to accountability mechanisms and budgetary allocations • Results-based planning and portfolio management mechanisms • New decentralized model using intermediate "value centers" that are more outcome-based, closer to the service/client interface (moving towards a demand-driven system)	• Use a budget system to link policies (suppliers and insurers), highlighted in the National Budget Report (system responsibilities and budget processes) • "Department of Finance" philosophy → macroeconomic forecasting of healthcare costs (not entirely a "budget") • VBTB (Policy Budgets and Policy Accountability, or in Dutch Van Beleidsbegroting Tot Beleidsverantwoording) is a national ministerial policy that links policy goals more explicitly to budgets and financial accountability. VBTB accelerates financial accounting and quality at the request of the Lower House of the Dutch Parliament. • Mixed tax-based and insurance financing • Currently modernizing and reforming the budgetary processes through portfolio management, CEA and cost-of-illness data

**Health system planning**	

• Ministry has devolved power for planning and coordinating local healthcare to the LHINs • 3 year planning reports fed by the OHSS and LHSS → Ministry still regulates planning at the LHIN level • LHINs can outsource services, engage the community, make proposals to the Ministry and integrate local services	• Ministry uses the 15 themes of the Zorgbalans for health system planning (eg. investments, wait times) • Zorgbalans to be used as a tool for increasing transparency (planning role and system responsibilities are presented in the Zorgbalans) • Zorgbalans to be used as an information base for evidence-based planning and decision making

### Harmonizing the HSPA frameworks

**Figure 3:**
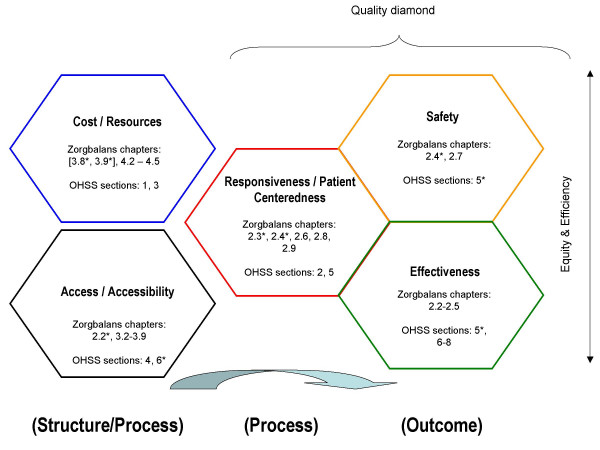
Harmonized Five-Diamond Framework.

In order to articulate the interrelations between the performance dimensions and corresponding indicator sets within and between each framework, we mapped the dimensional overlap between the two frameworks. This was used to develop a unified framework mechanism (Figure 3) to systematically link each system's overall aims, goals, performance measures and strategies embedded within each conceptual framework.

This backbone 'five diamond' framework merges the Dutch Zorgbalans healthcare performance matrix and OHSS framework, integrating Ontario's nine thematic areas (overall health system goals) within the broad consensus-based dimensions of the Dutch Zorgbalans' healthcare performance matrix.

This process involved integrating information and definitions from the CHIF, Zorgbalans framework, and OHSS. The performance matrix was selected to serve as the key theoretical base for several key pragmatic, functional reasons, one of which was to promote collaboration and common learning in both constituencies, and to expand this cooperative effort to other interested parties.

**Figure 4:**
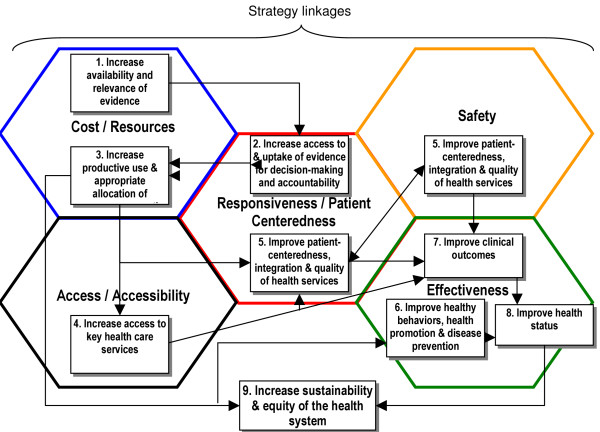
Incorporating Strategy into the Harmonized Five-Diamond Framework.

To further understand the interrelationships existing both within and between each framework, Ontario's Health System Strategy Map was embedded into the unified diamond framework (Figure 4). This step harmonized both frameworks for performance measurement, and illustrated how Ontario's hypothesized cause-and-effect linkages and strategy fit and interrelate within each system's performance dimensions – ultimately linking performance data with performance management and accountability.

## Discussion

Policymakers in Ontario and The Netherlands have expressed interest in and support for studies comparing their respective health systems performance assessment approaches, an important step providing a conceptual basis for any future benchmarking effort. Stakeholders representing the Dutch Ministry of Health, Welfare and Sport, University of Amsterdam Medical Center (AMC), and OMHLTC met in Toronto to promote such collaborative research and mutual learning.

We explored each constituency's conceptual HSPA framework, the embeddedness of performance data within management and policy functions, the extent of any overlap between the two frameworks, and relevant contextual factors that must be taken into account when comparing health system performance.

### Conceptual Issues

The Dutch framework governing the 2006 Zorgbalans is broad and comprehensive, composed of a large set of indicator areas that are relevant to the various departments at their MoH. The 3 chapters, 12 sub-dimensions, and 125 indicators of the Zorgbalans give a thorough review of areas relevant to the technical quality of healthcare in The Netherlands. However, the sheer complexity and number of performance indicators makes it difficult to identify performance areas requiring attention. The Netherlands should look at iteratively refining their indicator sets to provide a better picture of performance to policymakers.

The Zorgbalans fits well with the aims, goals and functions of the Dutch health system. As of January 2006, The Netherlands has changed its main steering philosophy from a budget-driven to a regulated market mechanism [3,21]. Given this steering philosophy, health system integration is not an explicit strategic priority or goal of the Dutch MoH. Therefore, the Zorgbalans does not explicitly link performance data to strategy and management functions. Rather, the onus is on each stakeholder to draw the conclusions they need from the Zorgbalans [21]. However, without embedding strategy, the current design does not make full use of available performance data.

The 2006 OHSS focuses on health system integration. [12]. Using its Health System Strategy Map, the Ontario Scorecard links measures, strategies, goals and outcomes, thereby enhancing accountability and assisting empirically sound evidence-based decision making across multiple sectors of the system [4,12,24]. However, the balanced set of 27 indicators is perhaps too restrictive and narrow to truly "best reflect the full extent of the health system's ongoing performance improvement initiatives".

The Netherlands and Ontario can build on each other's mix of performance indicator types in order to maintain a multi-stakeholder perspective, as different stakeholders have different views as to what processes and outcomes should be measured and how [25-27].

### Contextual policy factors

Researchers should also understand the higher-level contextual meaning behind selected benchmarking measures. The Ontario and Dutch healthcare systems, characterized mainly as Beveridge and Bismarckian systems, respectively, are undergoing great structural and regulatory changes. Ontario is currently transforming its healthcare system through decentralization/regionalization reforms aimed at health system integration and supply-side cost containment, whereas The Netherlands is pioneering a regulated-market steering philosophy focusing on demand-side rationing. Table 2 lists important policy context factors that must be taken into account when performing a benchmark.

The Zorgbalans' framework was designed to strategically fit with the new Dutch regulated-market steering philosophy focusing on demand-side rationing. The Dutch health system, mainly characterized as Bismarckian, is made up for four key sectors (public health, acute care, long-term care, and social care) that are regulated and financed through a mixture of private and public insurance schemes, along with municipal governmental budgets. Public and private sector actors have different roles in governing the healthcare sector. Municipalities are responsible for governing public health and social care (health), whereas private sickness funds are responsible for acute and long-term care sectors (healthcare) [28]. Due to the multitude of actors, each stakeholder is expected to draw relevant conclusions from the Zorgbalans, keeping overall health system targets in mind. System level accountability and transparency is to be managed through performance measurement, mainly focusing on suppliers and insurers, while maintaining a balance of mixed private sector and public finance.

Central to Ontario's decentralization reforms are the LHINs, not-for-profit corporation responsible for the planning, integration, and funding of local health services in fourteen geographic areas in Ontario. LHIN performance will be managed by cascading Ontario's performance Strategy Map to the local and provider level.

Such contextual information is necessary to understand the similarities and differences of their healthcare system approaches, along with the potential benefits and drawbacks of policies affecting the structure, design and organization and delivery of health services. Policymakers are interested in exploring novel regulatory regimes that encourage providers and patients to make choices that take both costs and outcomes into account. Canadian stakeholders are interested in learning from the "mixed market" models being used in Europe to determine how well they could serve the Canadian system.

### Comparing the performance frameworks

Conceptually, we demonstrate that it is possible to map the theoretical frameworks using a backbone 'five diamond' framework linking the Dutch Zorgbalans healthcare performance matrix and Ontario Scorecard. Figure 4 gives a clearer idea of the conceptual and contextual background of any performance dimensions and measures they intend to use in any future comparative project. Contextual policy factors were discussed in a workshop, giving clearer meaning to the comparative framework, and to stimulate ideas about how each constituency's regulatory model could serve towards mutual health system performance improvement:

This comparative study has policy implications and lessons for the development of future international collaborative benchmarking projects. The purpose behind this study is not to be overly prescriptive in the sense of pointing policymakers to a particular set of comparable indicators, but to articulate the interrelations between the performance dimensions and corresponding indicator sets within and between each framework. The onus is on them to then choose the indicators that fit their particular interests and policy priorities, and to understand their true contextual meaning within each constituency. Such a theoretically-sound empirical approach can help give a relatively objective view of performance over time and space, thereby providing the necessary evidence-base for actionable policy.

### Limitations

Considering the complexity of the topic, we acknowledge the shortcomings of being brief and abstract in each topic of discourse covered. HSPA is a dynamic field, and both the Dutch Zorgbalans and Ontario Scorecard are under continuous revision. Therefore information may and will change by the time this paper is published. We also acknowledge that certain assumptions and speculations were made when deriving the harmonized 'five diamond' framework, its performance dimensions and strategy linkages, all of which may be influenced by researcher and information bias. Much of the data received was in Dutch, and there is a possibility of information being lost in translation to English. Nevertheless, we attempted to be objective and thorough with our findings, towards giving researchers and policymakers the global bigger picture of comparative HSPA, in the hopes of stimulating future research and collaboration across the Atlantic.

## Conclusion

We compared health system performance management approaches in The Netherlands and Ontario, highlighting various conceptual and contextual policy factors that must be taken into account when attempting any future benchmark. Conceptually, it is possible to map both theoretical frameworks, as shown by a backbone 'five diamond' framework that details interrelations and overlap between their goals, themes, and performance dimensions. We argue that performance assessment can be much improved if dimensions and indicators are well defined and tied into each constituency's policy and management processes. The Netherlands and Ontario can build on each other's mix of performance indicator types to maintain a multi-stakeholder perspective. We also highlight important contextual policy factors that must be taken into account, in order to better understand the meaning of selected performance measures and to promote common learning about the potential benefits and drawbacks of policies affecting the structure, design and organization and delivery of health services in two constituencies using differing regulatory regimes.

## Competing interests

The author(s) declare that they have no competing interests.

## Authors' contributions

ART secured the funding, assisted in designing the study, performed the literature review and data retrieval, and drafted the manuscript. NSK conceived the study, assisted with study design, coordinated all study activities, and critically examined the manuscript for intellectual content. OAA helped design the study, and assisted with data interpretation, manuscript drafting, and final presentation of findings. All authors read and approved the final manuscript.

**Table 1:** 2006 Zorgbalans: Dutch health care performance report [21, 22]

**Chapter 2: what is the quality of the care?**	**Chapter 3: how accessible is the health care?**	**Chapter 4: how much costs the health care?**
The effectiveness of prevention (12 indicators, s, p, o*) The effectiveness of curative care services (20, p, o) The effectiveness of long-term care (8, o) The effectiveness of mental health care and substance abuse care (5, o) Consumer experiences with health care (2, o) Patient safety (6, p, o) Quality systems in health care (4, s) Innovation in health care (6, s, p)	Choice and access to care (2, p) Access to acute and life-saving care (5, o) Waiting times for regular care (4, o) Access according to needs (4, o) Financial access to care (8, o) Geographical access and regional distribution of care (2, o) Personnel and staffing (5, s, o) Health care professions and health care training (7, s)	Macro costs (10, s) The health care market (8, s) Labour productivity in health care (3, s, p) The financial position of care institutions (5, s)
Scope – 63 indicators, with a mix of s, p, o, within 8 themes	Scope – 37 indicators, mainly outcome, within 7 themes	Scope – 26 indicators, mainly structure, within 4 themes

* s – structure, p – process, o – outcome

## Pre-publication history

The pre-publication history for this paper can be accessed here:


