# Correction: Comparing health system performance assessment and management approaches in the Netherlands and Ontario, Canada

**DOI:** 10.1186/1472-6963-7-44

**Published:** 2007-03-27

**Authors:** Ali R Tawfik-Shukor, Niek S Klazinga, Onyebuchi A Arah

**Affiliations:** 1 Department of Social Medicine, Academic Medical Center, University of Amsterdam, PO Box 22700, 1100 DE Amsterdam, The Netherlands; 2 Netherlands Institute for Health Sciences, Erasmus MC, PO Box 2040, 3000 CA Rotterdam, The Netherlands

Following the publication of our recent work [1], we were asked by the *Zorgbalans *(Dutch Healthcare Performance Report) project at the National Institute of Public Health and the Environment (RIVM) to withdraw the RIVM affiliation of the third author (O.A. Arah) who is a member of the *Zorgbalans *project team from the paper. It should be clear that the paper was not – and is not – officially endorsed by the RIVM.

We regret any inconvenience this apparent oversight and inappropriate affiliation use might have caused. Dr. Arah partook in the paper in his capacity as a faculty member of the Department of Social Medicine at the Academic Medical Center of the University of Amsterdam. The study was funded by a specific third party Canadian grant to that department (see acknowledgement in the paper [1]).

In order to maintain our academic freedom and independence, we kindly ask the readers to disregard the aforementioned inappropriate affiliation and to consider this correction as a public withdrawal of the affiliation with the National Institute of Public Health and the Environment (RIVM) from the author byline.

We also wish to correct the oversight in the abstract and text of the paper that the Dutch performance framework focuses on technical quality. This is mistaken since the Dutch framework also focuses on access and cost (as was already mentioned in the paper).

As stated in the acknowledgement, the paper remains a property and responsibility of the authors and none of their employers, affiliate institutions, sponsor, interviewees or informants can be held responsible for its content and interpretations. We did/do not seek their approval of our interpretations nor should any such approval be ascribed to them.

## Pre-publication history

The pre-publication history for this paper can be accessed here:


